# EFL reading goals of grade 11 students across public and non-public schools in Addis Ababa, Ethiopia

**DOI:** 10.1016/j.heliyon.2017.e00396

**Published:** 2017-09-06

**Authors:** Tekle Ferede, B.M. Nchindila

**Affiliations:** Jimma University, Jimma 378, Ethiopia

**Keywords:** Education

## Abstract

This study investigated the EFL reading goals of Grade 11 students across public and non-public schools in the Ethiopian capital, Addis Ababa. To this end, quantitative data were collected from 556 (375 public and 181 non-public) students via pre-tested structured questionnaire and analyzed into means, medians, standard deviations, ranges and Mann-Whitney U test scores. The results show that non-public school students were found better than public school students in possessing components of both extrinsic and intrinsic goals for reading. The notable exception in this regard is that public school students had higher social motivation for reading than their non-public school counterparts. Based on this finding, it has been concluded that non-public school students have a better chance of evolving as persistent self-initiated EFL readers since they have various goals which urge them to engage in reading a range of texts. It is thus recommended that English language teachers in public schools should constantly take actions to enable their students to develop appropriate EFL reading goals.

## 1 Introduction

### 1.1 Background of the study

Students’ reading goals determine what they read, when they read, where they read, how frequently they read and how sustainable their habits can be. Reading goal embraces, among other things, intrinsic motivation, extrinsic motivation, goal orientation and values of reading (Watkins and Coffey, 2004). Students with intrinsic purposes become curious and interested in a learning activity for its own sake, not for the extrinsic incentives it elicits (Dornyei, 2005). For such students, the worth of reading takes precedence over the rewards that it generates. This urges them to engage in more self-initiated reading due to the desire to read about a subject of interest and the enjoyment they acquire from reading self-selected texts. On the other side, students with extrinsic goals read for external reasons such as good results, rewards or positive feedback (Lau, 2009). These students are characterized by the goal orientation of working mainly to perform well in the eyes of others. In effect, they tend to involve in competition, work to outperform others and struggle to win the recognition of other parties such as teachers (Baker and Wigfield, 1999).

Baker and Wigfield, based on the works of Baker et al. (1996) and Guthrie et al. (1996), also discuss social purpose for reading which draws on the notion of reading as a social activity. One point that can be raised in this connection is that social factors play an important role in students’ reading behaviour and achievement. A useful feature of social purpose for reading is the need for mutual construction of meaning from a text and sharing the information obtained from reading with their partners and family; the second aspect is known as compliance, i.e. reading to fulfill the expectations of others such as teachers, parents and so on (Wentzel, 1998; as cited in Baker and Wigfield, 1999).

Generally, whether students read to obtain satisfaction from reading, to get external benefits or to engage in cooperative meaning construction, the purpose for which they read determines their reading achievement, reading perseverance and choice of texts.This implies that reading goal is one of the issues that reading instruction should address. In addition, students’ goal orientation in reading and the instructional measures taken in dealing with reading goal orientaion need researching. This study was thus aimed to investigate Grade 11 students’ EFL reading goals across public and non-public schools in an Ethiopian context.

### 1.2 Statement of the problem

As highligted above, students with intrinsic purposes engage in reading for the enjoyment they acquire out of it. They are likely to engage in sustainable self-initiated reading regardless of the culmination of a language study programme (Gambrell et al., 1996). This means, they can become life-long independent readers, for the desire to read comes from within. On the other hand, learners with extrinsic purposes read to obtain external benefits (Baker and Wigfield, 1999). Recognition, grades and competition stand out as domains of their reading goal. In this case, reading is mostly done in the classroom where a student’s reading comprehension performance is assessed and compared with the performance of other students. While extrinsic goal can lead to successful reading achievement, it can cease with the completion of the language learning program or the withdrawal of rewards. Thus, it can have limitations in enabling students to become life-long independent readers.

In the Ethiopain context, English is used as a foreign language (EFL)with its function mainly limited to school classrooms. Students study English as a subject from Garde 1 and learn other subjects through this language in indifferent grade levels. In this case, there are concerns that students tend to read for extrinsic benefits such as obtining good results. With this reading goal, they are likely to cease reading EFL texts as they leave schools due to the terminations of their study programmes. This being the case, however, there are no published sources which examined the EFL reading goals of secondary school students in the Ethiopian context. Studies which compare public and non-public schools in the same context in terms of their EFL reading goals are equally absent.

Differences between public and non-public schools regarding students’ reading goals may seem obvious. However, what is commonly held can be misleading due factors such as school culture, teachers, instructional focus, textbooks, assessment methods, etc. These factors evidently differ from context to context. For example, the current Ethiopian Government makes huge investments to make long term and short term training opportunities available for EFL teachers at all levels. Nevertheless, this is not the case for non-public schools. In addition, continuous assessment is integral to the teaching-learning process in public schools while it may not be compulsory in non-public schools. These and other factors can bring about reading goal differences, differences that are not apparent, between public and non-public school students. This shows that empirical studies are necessary to find out differences in EFL reading goal between public and non-public school students. Therefore, this study tried to contribute to endeavours made to fill this gap. In effect, it investigated the reading goals of Grade 11 students across public and non-public schools in the city of Addis Ababa, Ethiopia. To achieve this aim, the study attempted to answer the following basic questions.

### 1.3 Research questions

The main research question posed in this study was “What are the EFL reading goal orientations of Grade 11 students across public and non-public schools?” The following, research sub-questions were then extracted from this main research question and eventually answered accordingly:

1. Which type of EFL reading orientation (intrinsic, extrinsic or social) is dominant among Grade 11 students across public and non-public schools?
2. Are their significant differences in EFL reading goals between Grade 11 students in public schools and those in non-public schools?

## 2 Materials and methods

### 2.1 Study setting

This descriptive-comparative survey was carried out on selected public and non-public schools in Addis Ababa, the capital city of Ethiopia. Addis Ababa, currently made up of 10 sub-cities, is a seat for various organizations including the African Union (AU), the headquarters of the United Nations Economic Commission for Africa (UNECA), the Federation of African Societies of Chemistry (FASC) and the Horn of Africa Press Institute (HAPI). The presence of these organizations in Addis Ababa has resulted in the launching and development of international private schools and foreign community schools in the city.

Since Addis Ababa is the commercial, diplomatic, political, religious and educational hub of Ethiopia, the first public and private schools emerged in this city. Both school systems expanded progressively especially after the coming-to-power of the current government in 1991. Private schools have grown in number at all levels under varying systems of ownership. Some private schools are owned by local investors. These schools, like public schools, use textbooks prescribed by the Ministry of Education (MoE) and mostly follow the directions and guidelines of the Ministry. In addition, they are similar with public schools in terms of resource provision and incentive allocation for teachers. In the second category are schools set up by religious societies, private schools run by foreign investors or Diaspora Ethiopians and community schools under foreign holding. Community schools mainly serve diplomatic communities, foreign nationals and Ethiopians who attained foreign citizenship. Schools that operate under the ownership of religious groups, in most cases, use English textbooks provided by MoE, and some supplement them with their own materials. On the contrary, international private schools and foreign community schools, which are better resourced and have better incentives for teachers, majorly use their own coursebooks.

Therefore, international private schools and foreign community schools have several characteristics in common. Firstly, both largely use their own coursebooks. Secondly, both are better resourced, have better remuneration for teachers and enroll students from rich families. Thirdly, most of the schools in both systems have students whom they prepare for international examinations such as the International Baccalaureate (IB) and TOEFL. Therefore, in this study, these schools are grouped together under the name ‘non-public schools’. On this basis, public schools and non-public schools were surveyed since the two systems vary in resources, choice of textbooks/coursebooks, incentives for teachers and students’ goals for learning English. On the other side, whereas the development of public schools and schools owned by local businesses in Addis Ababa has been accompanied by similar developments in other towns in the country, international private schools and foreign community schools have concentrated in Addis Ababa. This shows that Addis Ababa is the best site for a study that aims to compare Grade 11 students’ EFL reading goals across public and non-public schools.

### 2.2 Population and samples

This study focused on the population of Grade 11 students in public and non-public schools in Addis Ababa, who were enrolled in the 2015/2016 academic year. During data collection, there were 27 (19 public and eight non-public) preparatory schools (consisting of Grade 11 and Grade 12) in Addis Ababa. The non-public schools include international private schools and foreign community schools. In this study, covering all the 19 public and the eight non-public schools was not preferred since there can be “diminishing returns associated with adding elements to a sample” (Dattalo, 2008; p.7). Instead, sample schools were selected in the following ways.

The public preparatory schools were distributed across the 10 sub-cities of Addis Ababa. Since these schools were equivalent in terms of resource provision, teacher remuneration and student background, selecting sample schools from any of the sub-cities could have been fairly sound. However, taking account of the distribution of sample schools across a relatively large number of sub-cities was considered more reasonable. Accordingly, five sub-cities (50%) of the total were chosen by a lottery draw. Then, one preparatory school was included in the study from each selected sub-city. Where there were two or more preparatory schools in a sub-city, one was chosen using lottery draw. In this way, five public preparatory schools were sampled.

The selection of the sample schools was accompanied by sample size determination in line with the population of Grade 11 students in the 19 public schools (N = 14445). Accordingly, the sample size determination method proposed by Krejcie and Morgan(see Krejcie and Morgan, 1970; p.608) was followed. These scholars created a Table that can be used to know the sample size for a particular defined population without a need for any calculation. This Table is based on the assumption that “as the population increases the sample size increases at a diminishing rate and remains relatively constant at slightly more than 380 cases” (Krejcie and Morgan, 1970; p.607). It was constructed using the formula: *s* = X^2^NP(1-P) ÷ d^2^(N-1) + X^2^P(1-P) in which *s* = required sample size, *X*^2^ = Table value of chi-square for 1 degree of freedom at the desired confidence level (3.841), *N* = the population size, *P* = the population proportion (assumed to be .50 since this would provide the maximum sample size) and *d* = the degree of accuracy expressed as a proportion (.05).

According to Table values, the sample size for a population of 10000 is 370 while it is 375 for a population of 15000. The population of Grade 11 students in the 19 preparatory schools (N = 14445) was closer to 15000 than to 10000. Hence, a sample of 375 students was taken to represent this population. This procedure yielded a large-enough sample size (n = 375) which was distributed across the selected schools.

However, the non-public schools were not distributed across sub-cities with the same pattern as the public schools: some sub-cities did not have such schools at all. Thus, the selection of sample non-public schools was done in line with the population of Grade 11 students in these schools regardless of their locations in sub-cities.

Five international private schools and three foreign community schools constituted the category of non-public schools. The population of Grade 11 students in these schools during data collection was 343. In the Krejcie-Morgan table, it is shown that 181 samples are required for a population of 340 to which 343 is very close. Therefore, the sample size for non-public schools was determined to be 181. As the population sizes in such schools were small, maximizing the number of target schools was considered necessary. To this effect, three international private schools and two foreign community schools (N = 5) were chosen by lot to which the total sample was distributed.

At first, systematic random sampling was planned for recruiting each sample element from the respective school. However, it was abandoned during the actual sample selection due to sound reasons. The population sizes in the non-public schools were too small to lend themselves to systematic selection of samples. This called for the use of availability sampling technique to select samples from the schools chosen in this category. To be consistent in sample selection, this method was also applied in public schools. According to Dattalo (2008, p.6), “Availability sampling is a technique in which elements are selected because of their accessibility to the researcher”. Therefore, students who were available in their classrooms during data collection were taken randomly or totally (taking account of the required sample size).

### 2.3 Data collection instrument

Part of the Motivation for Reading Questionnaire (MRQ), developed by Wigfield and Guthrie, 1997 and later validated by Baker and Wigfield, 1999, Guthrie et al., 2000 and Wang and Guthrie, 2004, was used in this study. It consists of items aimed to measure students’ intrinsic motivation (12 items: curiosity = 5; involvement = 4; importance = 3), extrinsic motivation (14 items: grades = 4; recognition = 5; competition = 5) and social motivation (7 items). Therefore, a total of 33 items were included in the questionnaire to generate data on intrinsic reading goals, extrinsic reading goals and social purpose for reading. Accordingly, the items in the intrinsic motivation scale were meant to measure the participants’ intrinsic reading motivation from the perspectives of curiosity, involvement and perceived value of reading. On the other hand, the items in the extrinsic motivation component were used to measure the students’ extrinsic reasons for reading in relation to the sub-scales of recognition, competition and grades. Finally, the items constituting the social motivation part of the questionnaire were intended to assess the study participants’ social reasons for reading.

This questionnaire has five response options: *Never true of me (1)*, *Sometimes true of me (2)*, *Cannot decide (3)*, *Usually true of me* (*4)* and *Always true of me (5*) adopted from Oxford, 1999. Therefore, the study participants were required to respond to each item on this five-point Likert scale by putting a tick mark under the appropriate option. The administration of the questionnaire took place face-to-face where the participants were gathered in pre-arranged rooms. This helped to clarify certain points and to maximize response rate. As a result, all copies were properly completed and returned.

### 2.4 Methods of data analysis

To address the basic questions posed in sub-section 1.3, this study used quantitative data collected through structured questionnaire. Therefore, quantitative methods of data analysis were employed. In this analysis, the data were entered into EpiData and exported to Statistical Package for the Social Sciences (SPSS) version 20.0 for analysis into means, medians, ranges, standard deviations and Mann-Whitney U test scores. However, mean scores and Mann-Whitney test scores were used in the interpretation of the results.

### 2.5 Ethical considerations

In this study, care and responsibility was taken to meet ethical standards in dealing with human participants. Firstly, to provide proof for the legitimacy of the study, ethical clearance certificate was obtained from the University of South Africa (UNISA). Secondly, support letters were submitted to the administrators of the target schools, and the purpose, methods and procedures of the study were explained to them. After further discussions were held with these officials, the research project was granted permission. Subsequently, the purpose, methods and procedures of the study were explained to the participants. In addition, for the sake of anonymity, the names of the schools and their locations (sub-cities) were not specifically mentioned in the study. In addition, all the participants were assured that the data collected from them would be treated confidentially and that participation in the study was anonymous and voluntary. On this basis, participants aged ≥ 18 years gave informed consent by signing forms prepared for this purpose while permission was secured from the parents or guardians of participants aged < 18.

## 3 Results

Table 1 summarizes the results on the sub-scales of intrinsic motivation (curiosity, involvement and importance of reading), extrinsic motivation (grades, recognition and competition) and social motivation (social purpose for reading). Regarding curiosity as a component of intrinsic motivation, the mean and median scores for public school students were 2.48 and 2.40 with a standard deviation and a range of .91 and 4.0 respectively. On the other hand, the mean and median scores of curiosity for non-public school students were 3.45 and 3.40 with a standard deviation and a range of .77 and 4.0 respectively. The mean score for public school students (2.48) was a little below the expected mean (3.0) while the mean score for non-public school students (3.45) was above the same expected mean. This highlights that non-public school students scored better than public school students on the items measuring reading curiosity.

**Table 1:** Descriptive Statistics for Reading Goal by Type of School.

Sub-scale of Reading Goal		School	N	Mean	Mdn	Std.dev.	Range
Intrinsic Motivation	Curiosity	Public school	375	2.48	2.40	.91	4.0
		Non-public school	181	3.45	3.40	.77	4.0
	Involvement	Public school	375	2.57	2.50	.99	4.0
		Non-public school	181	3.08	3.00	1.03	4.0
	Importance	Public school	375	4.06	4.33	1.06	4.0
		Non-public school	181	4.05	4.00	.87	4.0

Extrinsic Motivation	Grade	Public school	375	3.12	3.25	.938	4.0
		Non-public school	181	3.46	3.5	.900	3.5
	Recognition	Public school	375	3.26	3.20	1.30	4.01
		Non-public school	181	3.92	4.0	.905	4.0
	Competition	Public school	375	3.41	3.60	.965	4.01
		Non-public school	181	3.43	3.60	.988	4.0

Social Motivation	Social purpose	Public school	375	3.44	3.42	.956	4.0
		Non-public school	181	3.02	3.0	.892	3.86

A Shapiro Wilk’s Test (p = .000) and a visual inspection of the histograms, normal Q-Q plots and box plots showed that the curiosity scores were not normally distributed for both public and non-public school students. A skewness of .439(SE = .126) and a kurtosis of −.459(SE = .251) for public school students and a skewness of −.368(SE = .184) and a kurtosis of .068(SE = .365) for non-public school students were observed. Although a log transformation was also conducted, the curiosity scores were still not normally distributed for both public and non-public school students. Thus, a non-parametric test statistics, the Mann-Whitney test, was run since the curiosity scores were not normally distributed for students in both school categories. The Mann-Whitney test revealed that the curiosity scores were significantly greater for non-public school students (Mdn = 3.45) than for public school students (Mdn = 2.40), *U* = 51,849.5, Z = 10.896, *n _non-public_* = 181, *n _public_* = 375, *P* = .000). That is, there were statistically significant differences between the two groups of students on the sub-scale of curiosity: non-public school students were significantly more curious readers than their public school counterparts.

Concerning involvement, the second component of intrinsic motivation, the results in Table 1 indicate that the mean and median scores for public school students were 2.57 and 2.50 with a standard deviation and a range of .99 and 4.0 respectively. The Table also shows that the mean and median scores of involvement for non-public school students were 3.08 and 3.00 with a standard deviation and a range of 1.03 and 4.0 respectively. Here, too, the mean score for public school students (2.57) was below the expected mean (3.0) while it was above the expected mean by .08 for non-public school students. This also implies that the latter were better than the former in their practice of involved reading.

For the measure of involvement, too, a Shapiro Wilk’s Test (p = .000) and a visual inspection of the histograms, normal Q-Q plots and box plots showed that the scores were not normally distributed for both public and non-public school students. Specifically, a skewness of .137(SE = .126) and a kurtosis of −.876(SE = .251) were observed for public school students while a skewness of −.108(SE = .184) and a kurtosis of −.733(SE = .365) were seen for non-public school students. A log transformation was also conducted, but still the scores of involvement were not normally distributed for both public and non-public school students. Therefore, a non-parametric test statistics, the Mann-Whitney test, was conducted. The Mann-Whitney test revealed that the scores of involvement were significantly greater for non-public school students (Mdn = 3.00) than for public school students (Mdn = 2.50), *U* = 41,725.5, Z = 5.147, *n _non-public_* = 181, *n _public_* = 375, *P* = .000). That means, non-public school students were significantly better (statistically significant difference observed) than public school students in their practice of involved reading.

The results pertaining to importance of reading, the third aspect of intrinsic motivation, depict that the mean and median scores for public school students were 4.06 and 4.33 with a standard deviation and a range of 1.06 and 4.0 respectively. It is also shown that the mean and median scores of importance of reading for non-public school students were 4.05 and 4.00 with a standard deviation and a range of .87 and 4.0 respectively. The results illustrate that the mean score for public school students (4.06) and the mean score for non-public students were nearly equal, both being above the expected mean (3.0). From this, it can be concluded that the students in both school categories had positive and comparable views on the value of reading.

A Shapiro Wilk’s Test (p = .000) and a visual inspection of the histograms, normal Q-Q plots and box plots showed that the scores on importance of reading were not normally distributed for both public and non-public school students. A skewness of −1.011(SE = .126) and a kurtosis of .057 (SE = .251) were found for public school students whereas a skewness of −.851(SE = .184) and a kurtosis of .398(SE = .365) were observed for non-public school students. A log transformation was also conducted, but still the scores of importance of reading were not normally distributed for both public and non-public school students. As a result, a non-parametric test statistics, the Mann-Whitney test, was conducted. The Mann-Whitney test revealed that there was no statistically significant difference between non-public school students (Mdn = 4.00) and public school students (Mdn = 4.33), *U* = 30,524, Z = -1.349, *n _non-public=_* 181, *n _public_* = 375, *P* = .177) in respect to their views about the importance of reading. Thus, the mean scores and the results of the Mann-Whiney test corroborate in demonstrating that the students in both school categories had comparable positive views on the importance of reading.

The first component of extrinsic motivation, the second sub-scale of reading goal, measured in this study was grade, i.e. students’ tendency of reading English texts in order to obtain good grades. The results in Table 1 indicate that the mean and median scores on the measure of grade for public school students were 3.12 and 3.25 with a standard deviation and a range of .938 and 4.0 respectively. The results in the same Table also indicate that the mean and median scores on the measure of grade for non-public school students were 3.46 and 3.5 with a standard deviation and a range of .900 and 3.5 respectively. It is thus noticeable that the mean score for public schools students (3.25) and the mean score for non-public school students (3.46) were both above the expected mean (3.0), but the mean score for non-public schools exceeds the one for public school students by .34. This suggests that although students in both school categories registered positive scores on the measure of grade, those in non-public schools seemed more serious about obtaining good grades by reading relevant texts.

Again, a Shapiro Wilk’s Test (p = .000) and a visual inspection of the histograms, normal Q-Q plots and box plots indicated that the scores on the measure of grades were not normally distributed for both public and non-public school students. A skewness of −.225(SE = .126) and a kurtosis of −.523(SE = .251) were observed for public school students while a skewness of −.360(SE = .184) and a kurtosis of −476(SE = .365) were found for non-public school students. A log transformation was also conducted, but still the scores were not normally distributed for both public and non-public school students. Therefore, a non-parametric test statistics, the Mann-Whitney test, was conducted. The Mann-Whitney test indicated that the scores on grades were significantly greater for non-public school students (Mdn = 3.50) than for public school students (Mdn = 3.25), *U* = 39455.5, Z = 3.842, *n _non-public=_* 181, *n _public_* = 375, P = .000). This thus implies that non-public school students were more serious than their public school counterparts (statistically significant differences observed) about reading texts in order to obtain good grades.

On the level of recognition, the second component of extrinsic motivation, the results in Table 1 show that the mean and median scores for public school students were 3.26 and 3.20 with a standard deviation and a range of 1.30 and 4.01 respectively. The same Table also displays that the mean and median scores for non-public school students were 3.92 and 4.0 with a standard deviation and a range of .905 and 4.0 in the stated order. In other words, the mean scores of recognition for both public school students (3.26) and non-public school students (3.92) were above the expected mean (3.0). However, the mean score of recognition for non-public school students was higher than that of public school students. Based on these facts, it is possible to conclude that while both public school students and their non-public school counterparts read materials written in English for the purpose of gaining recognition from other people, the latter appeared to be more recognition-seeking than the former.

A Shapiro Wilk’s Test (p = .000) and a visual inspection of the histograms, normal Q-Q plots and box plots showed that the scores of recognition were not normally distributed for both public and non-public school students. A skewness of −.239(SE = .126) and a kurtosis of −1.160(SE = .251) were observed for public school students while a skewness of −.693(SE = .184) and a kurtosis of −.211(SE = .365) were seen for non-public school students. Although a log transformation was also conducted, the scores were not normally distributed for both public and non-public school students. Thus, a non-parametric test statistics, the Mann-Whitney test, was conducted since the scores of recognition were not normally distributed for students in both school categories. The Mann-Whitney test revealed that the scores of recognition were significantly greater for non-public school students (Mdn = 4.0) than for public school students (Mdn = 3.20), *U* = 41915, Z = 5.257, *n _non-public_ =* 181, *n _public_* = 375, *P* = .000). This confirms the fact demonstrated by the mean scores that non-public school students were more recognition-seekers than public school students in their reading behaviour. In other words, a statistically significant difference was observed, favouring students in non-public schools, between the two groups of students in their scores on the extrinsic reading goal of recognition.

The third sub-scale of extrinsic motivation dealt with under the dimension of reading goal was competition. Table 1 displays that the mean and median scores of competition for public school students were 3.41 and 3.60 with a standard deviation and a range of .965 and 4.01 respectively. The same Table depicts that the mean and median scores of recognition for non-public school students were 3.43 and 3.60 with a standard deviation and a range of .988 and 4.0 respectively. Here, the mean score of competition for public school students (3.41) and the mean score of the same construct for non-public school students (3.43), both above the expected mean (3.0), were close to each other. This suggests that students in both school categories were comparably competitive in their reading behaviours.

For the competition component, too, a Shapiro Wilk’s Test (p = .000) and a visual inspection of the histograms, normal Q-Q plots and box plots indicated that the scores were not normally distributed for both public and non-public school students. A skewness of −.406(SE = .126) and a kurtosis of −.561(SE = .251) were found for public school students, but a skewness of −.537(SE = .184) and a kurtosis of −.178(SE = .365) were identified for non-public school students. A log transformation was also conducted, but still the scores of competition were not normally distributed for both public and non-public school students. Therefore, a non-parametric test statistics, the Mann-Whitney test, was conducted. This test revealed that the scores of competition did not differ significantly between non-public school students (Mdn = 3.60) and public school students (Mdn = 3.60), *U* = 33,290, Z = .276, *n _non-public_ =* 181, *n _public_* = 375, *P* = .783). This finding supports the fact which the mean scores suggested above, i.e. students in both school categories were comparable in possessing the reading goal of competition.

The third dimension of reading goal addressed in this study was social motivation (social purpose for reading). The results, as can be seen in Table 1, revealed that the mean and median scores of social purpose for public school students were 3.44 and 3.42 with a standard deviation and a range of .956 and 4.0 respectively. On the other hand, Table shows that the mean and median scores of social purpose for non-public school students were 3.02 and 3.00 with a standard deviation and a range of .892 and 3.86 respectively. It is noticeable here that the mean score for public school students (3.44) was above the expected mean (3.0) while that of non-public school students (3.02) was nearly equal to the expected mean. That is, public school students had a greater mean score of social purpose for reading than non-public school students.

A Shapiro Wilk’s Test (p = .000) and a visual inspection of the histograms, normal Q-Q plots and box plots showed that the scores of social purpose for reading were not normally distributed for both public and non-public school students. A skewness of −.288(SE = .126) and a kurtosis of −.682(SE = .251) were identified for public school students while a skewness of −.005(SE=.184) and a kurtosis of −.566(SE = .365) were found for non-public school students. Although a log transformation was also conducted, the scores were still not normally distributed for students in both school categories. Consequently, a non-parametric test statistics, the Mann-Whitney test, was conducted. This test unveiled that the scores of social motivation for reading were significantly greater for public school students (Mdn = 3.42) than the scores for non-public school students (Mdn = 3.00), *U =* 24451.5, Z = -4.821, *n _non-public_ =* 181, *n _public_* = 375, *P* = .000). In other words, the scores on social purpose for reading revealed a statistically significant difference between public and non-public schools students in favour of the former.

## 4 Discussion

Motivated students have a wide range of personal goals for reading such as curiosity, involvement, social interchange and emotional satisfaction (Mohammad, 2017). In this study, reading goal was treated as subsuming intrinsic motivation (curiosity, involvement and importance of reading), extrinsic motivation (grades, competition and recognition) and social motivation (social purpose for reading). EFL reading goal was thus measured based on these components as indicators. Other factors such as reading competence and reading efficacy are addressed in another article.

With regard to intrinsic motivation, the results of the study demonstrated that public school students had mean scores lower than the expected mean (3.0) on the curiosity and involvement components while non-public school students had mean scores above the expected mean (statistically significant differences in favour of non-public schools observed). However, both public and non-public school students had comparable positive views on the importance of reading. On the extrinsic motivation sub-scale, on the other hand, students in both school categories registered positive scores on the measures of grade, competition and recognition. Nevertheless, while both groups had comparable inclinations towards competition, non-public school students had significantly better scores for the extrinsic goals of grade and recognition. Concerning social motivation, public school students had a greater mean score than non-public school students (statistically significant difference in favour of public school students identified). This can be the result of a systematic attention given to cooperative learning and its implementation in public schools.

The results thus suggest that non-public school students had more intrinsic (curiosity and involvement) and extrinsic (grades and recognition) goals for reading than public school students. From intrinsic motivation point of view, this means that they were better in curious reading (reading to quench one’s curiosity to learn new things) and involved reading (reading with immersion) which help them to develop independent reading habits for “… the more one reads, the better reader one becomes” (Gambrell, 2011; p.5). On the extrinsic side, non-public school students also appeared more serious about obtaining good grades (which is normally possible through reading) and demonstrated more recognition-seeking behaviour (reading texts with the intention to get recognition from teachers, parents and other people). These can be the results of stronger competition among non-public school students for grades and recognition than among their public school counterparts. It is also possible to speculate that students in non-public schools generally came from families with higher social status or higher income which helped them to develop a variety of intrinsic and extrinsic purposes for EFL reading. On the contrary, public school students excelled their non-public school counterparts in their score of social motivation (reading cooperatively with others). Here, it can be expected that their social purpose for reading, if put into effect, can help public school students to learn and apply components of social strategy more efficiently.

Studies established that reading motivation is a multi-faceted issue. For example, Komiyama (2013) found that adult English for Academic Purpose (EAP) students’ (in the USA) reading motivation had intrinsic and extrinsic dimensions. Similarly, a study conducted by Kim and Choi (2014) identified that Korean high school students’ ESL motivation was affected by several factors suggesting the multidimensionality of reading motivation. However, based on the findings of several studies (e.g. Gottfried, 1990; Wigfield and Guthrie, 1997; Guthrie et al., 2000; Vansteenkiste et al., 2004; Guthrie et al., 2007), Gambrell (2011) reports that intrinsic motivation can result in better reading achievement than extrinsic motivation. Students with intrinsic goals engage in reading for the satisfaction they acquire from it, not for temporary external rewards. Thus, other things being equal, they can become life-long independent readers for the desire to read which emerges from within is likely to develop into an enduring habit. Accordingly, non-public school students who possess better intrinsic motivation are likely to become better independent readers. On the other hand, public school students’ better social motivation can enable them to set mutual reading goals, give and receive feedback, monitor their reading comprehension and evaluate their reading progress in their cooperative reading efforts (Schrader et al., 2012). These practices, if enduring, can eventually help them to progressively move away from the direct control of the teacher and develop self-initiated reading habits.

## 5 Conclusion

The results of this study indicated that while students in public schools inclined more towards extrinsic goals and social purpose for EFL reading, those in non-public schools had a variety of both extrinsic and intrinsic reasons for the same (Research Question 1). On the other hand, significant differences were identified in most dimensions of reading goal, in favour of non-public school students, between the two groups of study participants (Research Question 2). It can thus be concluded that non-public school students, by virtue of more EFL reading goals, seem to have a better chance to emerge as self-initiated EFL readers. On the contrary, although public school students’ social purpose for reading can somewhat help them to develop self-initiated reading habits, they still appear to possess deficient intrinsic goals for EFL reading.Therefore, English language teachers in public schools should devise and implement mechanisms to enhance their students’ intrinsic motivation for EFL reading.

## Declarations

### Author contribution statement

Tekle Ferede: Conceived and designed the experiments; Performed the experiments; Analyzed and interpreted the data; Wrote the paper.

BM Nchindila: Contributed reagents, materials, analysis tools or data.

### Funding statement

This work was supported by Jimma University and the University of South Africa.

### Competing interest statement

The authors declare no conflict of interest.

### Additional information

No additional information is available for this paper.
